# Supplementary material for: COZOID: contact zone identifier for visual analysis of protein-protein interactions
Source: BMC Bioinformatics. 2018 Apr 6;19:125. doi: 10.1186/s12859-018-2113-6 (PMC5889581; doi:10.1186/s12859-018-2113-6)
Supplement: Supplementary file 2 — Software build. Executable binary file of the software tool. (ZIP 118,784 kb) [file 12859_2018_2113_MOESM2_ESM.zip › cozoid/platform/docs/testng-6.5.1-javadoc.zip!/org/testng/IMethodSelectorContext.html]

IMethodSelectorContext (TestNG)


---


|  |  |  |
| --- | --- | --- |
| **Overview** \| **Package** \| **Class** \| **Tree** \| **Deprecated** \| **Index** \| **Help** | |  |
| **PREV CLASS**   **NEXT CLASS** | **FRAMES**    **NO FRAMES**     **All Classes** |  |
| SUMMARY: NESTED \| FIELD \| CONSTR \| METHOD | DETAIL: FIELD \| CONSTR \| METHOD |  |


---


## org.testng Interface IMethodSelectorContext

---

``` public interface IMethodSelectorContext ```

An implementation of this interface is passed to all the Method Selectors
when their includeMethod() is invoked. Method selectors can invoke
any method of this context at that time.
Created on Jan 3, 2007

**Author:**
:   Cedric Beust

---

| **Method Summary** | |
| --- | --- |
| `java.util.Map<java.lang.Object,java.lang.Object>` | `getUserData()` |
| `boolean` | `isStopped()` |
| `void` | `setStopped(boolean stopped)`             Indicate that no other Method Selectors should be invoked after the current one if stopped is false. |

**Method Detail**

### isStopped

```
boolean isStopped()
```

:   **Returns:**: true if no more Method Selectors should be invoked after the current one.

---


### setStopped

```
void setStopped(boolean stopped)
```

:   Indicate that no other Method Selectors should be invoked after the
    current one if stopped is false.

    :   **Parameters:**: `stopped` -

---


### getUserData

```
java.util.Map<java.lang.Object,java.lang.Object> getUserData()
```

:   **Returns:**: a Map that can be freely manipulated by the Method Selector. This can be used to share information among several Method Selectors.


---


|  |  |  |
| --- | --- | --- |
| **Overview** \| **Package** \| **Class** \| **Tree** \| **Deprecated** \| **Index** \| **Help** | |  |
| **PREV CLASS**   **NEXT CLASS** | **FRAMES**    **NO FRAMES**     **All Classes** |  |
| SUMMARY: NESTED \| FIELD \| CONSTR \| METHOD | DETAIL: FIELD \| CONSTR \| METHOD |  |


---
